# Piece of Cake: Coping with COVID-19

**DOI:** 10.3390/nu12123803

**Published:** 2020-12-11

**Authors:** Melissa J. Chee, Nikita K. Koziel Ly, Hymie Anisman, Kimberly Matheson

**Affiliations:** 1 Department of Neuroscience, Carleton University, 1125 Colonel By Drive, Ottawa, ON K1S 5B6, Canada; nikitakozielly@cmail.carleton.ca (N.K.K.L.); hymie.anisman@carleton.ca (H.A.); kim.matheson@carleton.ca (K.M.); 2 Royal Ottawa Institute of Mental Health Research, 1145 Carling Avenue, Ottawa, ON K1Z 7K4, Canada

**Keywords:** stress, food choice, coping, COVID-19, snack, employment, mental health, salty, sweet, whole food

## Abstract

To limit the spread of coronavirus disease 2019 (COVID-19), many individuals were instructed to stay at home, and teleworking became commonplace. Meanwhile, many others were laid off or worked reduced hours, and some front line workers were required to work longer hours. Concurrently, a surge in reports of “pandemic baking” suggested a cascade effect on eating behaviors, which may be an inadvertent strategy to cope with stress. We conducted an online survey of people living in Canada or the United States (*N* = 680) to assess how employment change may have been experienced as stressful and linked to a shift in food choices. Regression models suggested that reduced hours and being laid off were associated with greater stress appraisals, avoidant- and emotion-focused coping responses, and negative affect. In turn, negative affect was associated with eating to cope and unhealthy snack choices, like salty or sweet treats. Our study emphasizes that under stressful conditions, such as those experienced during the COVID-19 pandemic, some coping strategies may contribute to the greater vulnerability to downstream effects, particularly those relating to eating choices and nutritional balances.

## 1. Introduction

In order to limit the spread of the coronavirus disease 2019 (COVID-19), countries around the world implemented public health measures, including physical distancing requirements to prevent close contact between people. These measures resulted in various home confinement directives comprising stay-at-home orders, isolation requirements, lockdowns that prohibit normal daily activities, such as going to school or work, visiting gyms or restaurants, or large social gatherings. During this time, many individuals transitioned rapidly to working from home where possible, but many others suffered job loss or worked fewer hours, while those on the front lines worked more and longer hours [1,2]. Accordingly, in addition to coping with the immediate health threat of COVID-19, individuals also coped with abrupt lifestyle adjustments as a result of employment change, often leading to economic hardships, as well as home isolation [3]. These sudden lifestyle changes frequently manifested as psychosocial stressors with negative consequences for mental health [4,5], including increased reports of depression and anxiety during the first wave of COVID-19 transmission [6,7,8,9,10,11]. Importantly, the combination of lifestyle and psychosocial stressors can be detrimental for body weight management, which can influence metabolic syndrome and overall physical health [12].

Nearly one in four adults recruited through social media or paid advertisements reported gaining up to 10 pounds during the first wave of COVID-19 [13], and weight gain was more prevalent in individuals with obesity [11]. This is particularly significant as two in three adults in North America are overweight or have obesity [14,15,16], thus, a sustained period of weight gain during the pandemic could further aggravate metabolic syndrome [6]. This has implications for related health conditions like obesity [6,17] and its comorbid diseases, including diabetes [18], hypertension [18], and cardiovascular disease [19] that are significant risk factors for COVID-19 severity [20]. Indeed, half of patients hospitalized for COVID-19 have obesity [21], and patients with obesity are also more likely to require a ventilator [22]. Even among younger patients, who are a lower risk group for complications related to COVID-19 infection, those who have obesity were twice as likely to require intensive care [23], and the severity of COVID-19 infection increased with greater obesity [22,23].

Healthy eating behaviors are essential features for body weight management [24], which is relevant for resistance to severe COVID-19 outcomes [25]. However, healthy eating can be disrupted when individuals are stressed. Conscious eaters who follow a restricted diet may report eating more when feeling stressed, and they are typically eating more highly palatable snack-type foods [26], particularly those high in fat, sugar, or salt, whereas consumption of fruit or vegetables may be reduced [26,27]. Furthermore, as comfort eating is a form of emotion-focused coping [28,29], experiencing negative affect or mood is also strongly associated with unhealthy snacking or binge eating [30,31,32]; conversely, positive affect or mood has been associated with healthy snack choices [33]. In effect, comfort eating can help individuals cope with negative moods and stress, essentially serving as a form of self-therapy, which may be detrimental in the long-term.

Individuals experienced greater feelings of depression or anxiety during the lockdown phase of COVID-19 [6,7], and the vast majority of research surveys completed during this time reported that individuals ate more and snacked on calorie-dense foods between meals [3,7,34,35,36,37]. This said, one study reported healthier eating because of reduced access to fast foods and preparing meals at home [38], and individuals consuming homemade meals were less vulnerable to unhealthy eating [36]. Aside from increased eating being tied to loneliness or boredom [39], comfort eating can help individuals cope with other ongoing stressors, such as those created by unemployment and financial hardships stemming from COVID-19 [3,28,36]. Job insecurity and job loss are strongly associated with poor mental health outcomes [40,41,42,43,44] and were widespread as a result of home confinement directives to contain the spread of COVID-19 [1,2]. While emerging studies revealed poor mental state and unhealthy eating among individuals during the COVID-19 pandemic, it is uncertain whether a relationship exists between negative mood and food choice or how coping with employment change may impact mood and/or food choice.

It has been proposed that, in response to stressful situations, individuals make appraisals concerning the threat that these challenges represent followed by secondary appraisals concerning their ability to contend with these threats [45]. These appraisals then promote the adoption of particular coping strategies that may vary across individuals depending upon previous experiences and dispositional characteristics [46]. These coping processes may, in turn, be related to mood alterations. In the context of the distress related to the COVID-19 pandemic, it is conceivable that these coping changes might also contribute to the eating patterns individuals adopted, including their propensity to snack on comfort foods. While early findings during the first wave of COVID-19 indicated that home confinement led to unhealthy snacking, the mechanisms responsible for this relationship are not known. 

In order to evaluate the relationships underlying coping mechanisms that impact mood and food choice, we conducted an online research survey during the COVID-19 pandemic to assess stressor appraisals, affective state, and snacking behavior in participants living in Canada or the United States. We hypothesized that:

1. Changes of employment status in the context of COVID-19 would be appraised as both stressful and uncontrollable.
2. Stressor appraisals would mediate the relations between change of employment status and greater use of particular coping strategies, including problem-focused, emotion-focused, and avoidant coping.
3. Changes of employment status, appraisals of the COVID-19 situation, and coping strategies would be predictive of positive and negative affect.
4. Negative affect, but not positive affect, would be associated with eating to cope, as well as unhealthy snacking behaviors, i.e., eating more salty and sweet processed snacks rather than wholesome/unprocessed foods.
5. Eating to cope would mediate the relations between negative mood and snacking behaviors.

We found that employment change increased appraisals of stress leading to worsened mood. This association is important because participants were eating to cope with stress and choosing unhealthy snacks like salty and/or sweet treats.

## 2. Materials and Methods

### 2.1. Participants and Procedure

We recruited participants via social media postings on Facebook, Instagram, and Twitter; email and snowball sampling; and Amazon Mechanical Turk (MTurk) to complete a 30-min survey on food choices, stressor appraisals, and coping strategies during the COVID-19 pandemic (Table S1). All participants were 18 years of age or older, living in Canada or the United States, and read and understand English. Participants recruited via MTurk were required to meet a Human Intelligence Task (HIT) approval rate equal or greater than 95% (i.e., the proportion of completed tasks previously approved). 

The survey was completed by a total of 781 participants between 22 May 2020 and 13 June 2020. However, 12.9% of completed surveys (*n* = 101; Table S2) were excluded because they were completed in less than 5 min; selected *No* for response effort (i.e., I provided honest, high-quality answers to the survey questions); provided height and/or weight values that resulted in a Body Mass Index (BMI) of less than 15 or greater than 55; did not complete 100% of questions; selected Other when asked how their employment status changed due to COVID-19. The final sample comprised 680 participants (Table 1) who were predominantly female, White, less than 29 years old (mean, *M* = 36.9 ± 14.7), and a mean BMI of 25.5 ± 5.8 kg/m^2^. The majority of participants had some post-secondary education and were in shared living arrangements. The majority lived in a city that implemented home confinement directives and were teleworking.

The survey was administered via Qualtrics (Provo, UT, USA). Participants accessed the survey and provided informed consent to voluntarily participate. The survey included questionnaires to assess demographics, COVID-19 experiences, food and beverage consumption, stressor appraisals, mood, and coping profile. At the end of the survey, participants received a debriefing form that included contact information and additional resources if they had questions or felt distressed after answering questions related to their mood and ability to cope. Upon survey completion, participants could choose to receive a $5 CAD electronic gift card to either Amazon.ca, Second Cup, or Starbucks. MTurk participants were compensated ($3 USD) by fund transfer into their MTurk account. This study was completed in accordance with the Declaration of Helsinki, and this protocol was approved by the Carleton University Research Ethics Board B (Clearance #112909).

### 2.2. Measures

#### 2.2.1. Demographics

Demographic questions included age, height, weight, gender, cultural affiliation, relationship status, household income, employment status, national location, living arrangement, and highest level of education completed. We calculated BMI by dividing the height of the participant in kilograms by the square of their height in meters as a measure of body fat; both height and weight were self-reported. Participants selecting *Other* as their current employment status included current university students and individuals on leave (e.g., maternity, sick, disability). Educational status was determined as the highest level of education completed (Table S3). Those with high school or less may have no or some high school completed or a high school diploma. Those with post-secondary education may have college/university credit; trade/technical/vocational training; or an Associate, Bachelor’s, Master’s, Professional, or Doctorate degree. 

#### 2.2.2. COVID-19 Experiences

We assessed whether COVID-19 impacted the health, employment, and lifestyle of our participants via four questions. We assessed whether they received a COVID-19 diagnosis (i.e., Were you or anyone you know diagnosed with COVID-19?), lived in a city under home confinement directives (i.e., Did your city implement a stay-at-home order?), experienced a change in employment status (i.e., How did your employment status change as a result of COVID-19?), or worked from home (i.e., If you were working during the COVID-19 pandemic, did you work from home or did you leave home to work?).

#### 2.2.3. Food and Beverage Consumption

Participants responded to the Beverage and Snack Questionnaire 2 (BSQ2) [47] to indicate how frequently over the past week did they consume 14 types of beverages and 10 types of snacks on a seven-point rating scale (frequency): 1 (never or less than 1 per week), 2 (1 per week), 3 (2–4 per week), 4 (5–6 per week), 5 (1 per day), 6 (2–3 per day), 7 (4+ per day). They were grouped by snack type: salty (i.e., low-fat or non-fat potato chips, tortilla chips, and corn chips; regular potato chips, tortilla chips, corn chips, and puffs; other salty snacks); sweet (i.e., candy, including chocolate, candy bars, jelly beans, hard candies, and gummies; doughnuts, pop tarts, croissants, pastries; cookies, brownies, pies, cakes; low-fat or non-fat frozen desserts; regular ice-cream and milkshakes); wholesome (i.e., vegetables; fruits). Scores for each group were averaged. Inter-item reliabilities were predictably low (Cronbach’s α: salty = 0.57; sweet = 0.61; wholesome = 0.74). Although the types of snacks were grouped based on content, eating one snack (e.g., non-fat potato chips) was not necessarily associated with eating another snack within the same type (e.g., regular potato chips).

#### 2.2.4. Mood

Participants responded to 20 mood adjectives in the Positive and Negative Affect Schedule (PANAS) [48] using a five-point scale ranging from 1 (not at all) to 5 (extremely) to describe how they felt at the moment. Positive affect was obtained by a combined total of scores for ten terms: active, alert, attentive, determined, enthusiastic, excited, inspired, proud, strong (Cronbach’s α = 0.90). Negative affect reflected the summed response to ten terms: afraid, ashamed, distressed, guilty, hostile, irritable, jittery, nervous, scared, upset. (Cronbach’s α = 0.92).

#### 2.2.5. Stress Appraisals

Participants responded to the 28-question Stress Appraisal Measure (SAM) [49] on a five-point scale ranging from 1 (not at all) to 5 (extremely) indicating their thoughts in that moment on various aspects of their situation during the COVID-19 pandemic. There were seven categories of stress appraisals, including threat, challenge, centrality, controllable by self, controllable by others, uncontrollable, and stressfulness. A principal components analysis of the means of these seven subscales indicated two components. Based on factor loadings derived from a varimax rotation, two appraisal dimensions were formed. One dimension reflected appraisals of the stressfulness of the COVID-19 situation; this was derived by averaging scores for threat, centrality, uncontrollable by anyone, and stressful as scored by SAM (Cronbach’s α = 0.86). Appraisals of the controllability of the COVID-19 situation were derived by averaging scores for challenge, controllable by self, and controllable by others (Cronbach’s α = 0.74). 

#### 2.2.6. Coping Strategies

The reduced version of the Survey of Coping Profile Endorsed (SCOPE) [46] comprised 27 questions to assess strategies to deal with problems or stressful situations in the past week. These questions reflected 14 specific coping strategies, which in turn formed three general coping styles: problem-focused coping (i.e., active distraction, cognitive restructuring, problem solving); emotion-focused coping (i.e., blaming others, self-blame, rumination, emotional expression, seeking social support); and avoidant coping (i.e., emotional containment, cognitive distraction, passive resignation, wishful thinking, humor). Scores for each of the specific strategies forming the general coping styles were averaged (Cronbach’s α for problem-focused coping = 0.71; emotion-focused coping = 0.79; avoidance coping = 0.76).

In addition, questions to assess coping by eating (i.e., I craved food; I ate, even when I am not hungry; I ate more; I ate less) were included. A mean response score for eating as a coping strategy was calculated, but did not include ratings of ate less, as this behavior was not correlated with the other items (Cronbach’s α = 0.89). 

### 2.3. Statistics

Preliminary analyses were conducted to examine the distribution of COVID-19 experiences. As changes of employment status demonstrated the greatest variability, the relations between change of employment status and demographic characteristics of the sample as well as difference in the model variables of interest (using one-way analyses of variance (ANOVAs) and chi-square (χ^2^) statistics, as appropriate). Significant ANOVAs were followed up with post hoc pairwise comparisons (Tukey’s HSD, *p* < 0.05). Continuous variables including age and income were analyzed with an ANOVA while gender, education, living arrangement, and national location were analyzed with a chi-square test.

To assess whether change of employment status was associated with participants’ appraisals of their situation and coping with stressors encountered during the COVID-19, a series of mediation models was conducted wherein change of employment status was the predictor, stress, and controllability appraisals the mediators, and each of the coping strategies used in the past week as the outcome variables. Employment status was treated as a multi-categorical variable, with the degrees of freedom divided using Helmert contrasts (X1: no change coded −0.75 vs. the other three forms of change were each coded 0.25; X2: no change coded 0, more hours −0.667, reduced hours/laid off coded 0.333; X3: no change/more hours coded 0, reduced hours coded −0.5, laid off coded 0.5). The PROCESS macro (Version 3.3) applying model 4 [50] was used. The macro was set to use bootstrapping procedures with 5000 resamples. Moderated mediation analyses (applying model 8 of the PROCESS macro, which evaluates whether moderation occurred in the relation between the predictor and mediating variables or in the direct relation between the predictor and outcome variables [50]) were conducted to assess whether the relations between employment status, stressor appraisals, and coping were moderated by demographic characteristics of participants. Significant interactions were followed up with simple slope analyses conducted at 1 *SD* below and above mean of the relevant moderating variable. The 95% confidence interval (CI_.95_) is reported where applicable.

To assess whether change of employment status was associated with affective states, and whether consideration of stress appraisals and coping contributed additional understanding of affective outcomes, hierarchical linear regression analyses were conducted to predict positive and negative affect (separately), entering the three contrasts reflecting employment change, followed by stress and controllability appraisals, and finally the three coping styles. The moderating role of demographic variables was subsequently assessed by including their main effects and two-way interactions (cross-products) between the moderator and each predictor; separate analyses were conducted for each moderating variable.

After examining correlations to determine whether appraisals and coping were associated with eating choices, and whether the associated affect might be an important predictor of eating to cope, a series of mediation models was conducted wherein affect was the predictor (positive and negative affect in separate models), eating to cope as the mediating variable, and each of the snacking choices as the outcome variables. The PROCESS macro (Version 3.3) applying model 4 [50] was used, and the moderating effect of change of employment status and the demographic variables were then assessed (using the PROCESS macro model 8).

## 3. Results

### 3.1. What Was the Variation of COVID Experiences, and the Associations between Such Experiences, Demographic Features of the Sample, and the Model Variables of Interest?

#### 3.1.1. COVID-19 Experiences

Very few participants were exposed to COVID-19 directly, with only one participant indicating a positive diagnosis, another 28 (4.1%) reporting symptoms but had not been tested, and 20 (2.9%) had someone close to them that was positively diagnosed. Almost all participants (*n* = 623, 92.2%) lived in a city with a stay-at-home order, and of those who were employed, 76.2% worked from home. The employment status of just over half of the sample (*n* = 389, 57.2%) remained the same since the outbreak of COVID-19. Of these participants, 266 (68.3%) were employed, whereas 65 (16.7%) were unemployed, and 51 (13.7%) retired; there were no significant differences associated with the employment status of this subset of the sample on any of the model variables in the present study, and so analyses of change of employment status combined them in the no change category. Of those whose employment status changed, 34 (5.0%), worked more hours, 97 (14.3%) worked reduced hours, and 160 (23.5%) were laid off. As most of the variability associated with COVID-19 stressor experiences was a function of employment status change, we used this as our independent variable to assess the COVID-19 stress response.

#### 3.1.2. Relations between Change of Employment Status Due to COVID-19 and Demographic Features

As shown in Table 2, changes of employment status varied in relation to participant age (η^2^ = 0.093, *F*(3676) = 23.49, *p* < 0.001) and family income (η^2^ = 0.022, *F*(3673) = 5.01, *p* = 0.002). Specifically, those who did not experience employment changes tended to be older. As seen in Table S4, there were variations among those who did not experience a change of employment following the COVID-19 physical distancing measures. While a sizable proportion of the sample had been previously employed, others were already unemployed or retired. These subgroups further varied, in that, not surprisingly, those who retired were older (F(3385) = 124.88, *p* < 0.001). Those who were employed were more likely to be men (χ^2^(3) = 9.34, *p* = 0.025), living with children (χ^2^(9) = 52.85, *p* < 0.001), and living in the US (χ^2^(3) = 20.14, *p* < 0.001). In contrast, those who were already unemployed had less education (χ^2^(3) = 26.55, *p* < 0.001) and were more likely to be living with others and in Canada.

Younger, lower income participants were more likely to be laid off or to have their hours reduced. Increased work hours were more likely to be experienced by those with higher income. Employment change further varied with gender (χ^2^(3) = 16.59, *p* = 0.001), education (χ^2^(3) = 33.56, *p* < 0.001), living arrangement (χ^2^(9) = 35.29, *p* < 0.001), and national location (χ^2^(3) = 35.69, *p* < 0.001). As shown by the frequency distributions in Table 2, women, those with less education, and Canadians were more likely to be laid off. Those living alone were least likely to experience employment changes. In this particular sample, the participants living alone with children were all women (*n* = 21), and half of these women experienced no change of employment (*n* = 11), whereas a third (*n* = 8) had reduced hours or were laid off.

#### 3.1.3. Model Variables

As seen in Table 3, change of employment status had implications for appraisal variables, coping variables, and affect. On the whole, no change of employment status was associated with lower stress appraisals, less need to cope using any strategy, lower negative affect, and less snacking (specifically on salty foods). In contrast, being laid off was associated with higher scores on each of these variables.

#### 3.1.4. Summary

Employment change was associated with several demographic variables, with younger, lower income, females in shared living arrangements, and living in Canada being most likely to be laid off. Being laid off was also related to variables suggesting that these participants experienced greater distress, as they reported greater stress appraisals, more coping efforts, and negative affect but not necessarily poorer eating choices. 

Consideration was given to whether employment change was confounded with demographic variables, or whether they ought to serve as covariates in our main analyses. Indeed, younger participants were more likely to engage in salty (*r* = −0.17, *p* < 0.001) and sweet snacking (*r* = −0.13, *p* = 0.001), as were males (salty, *r* = 0.22, *p* < 0.001; sweet, *r* = 0.12, *p* = 0.003) and those living in the United States (salty, *r* = 0.15, *p* < 0.001; sweet, *r* = 0.11, *p* = 0.004). Conversely, compared to those living with others (with or without children) were more likely to snack on salty foods compared to those living without other adults (η^2^ = 0.012, *F*(3676) = 2.85, *p* = 0.037). BMI was considered in relation to snacking choices but was only mildly significantly related to being less likely to snack on wholesome foods (*r* = −0.08, *p* = 0.047). As one of the assumptions of covariance is that covariates are not related to the predictor variable (change of employment status) in a systematic way, these demographic characteristics were not deemed appropriate as covariates. Moreover, multivariate ANOVAs were conducted to assess the relations between employment change and each of the variable sets (i.e., snack choices, appraisals, coping, and mood) controlling age, gender or location. In all instances, variations associated with employment change remained significant, suggesting that these differences were not confounded with demographic characteristics. However, we assessed their moderating roles on the relationships among model variables, which were reported for each set of analyses.

### 3.2. Were Changes in Employment Status as a Result of COVID-19 Associated with Stressor Appraisals and General Coping Strategies?

**Figure 1:**
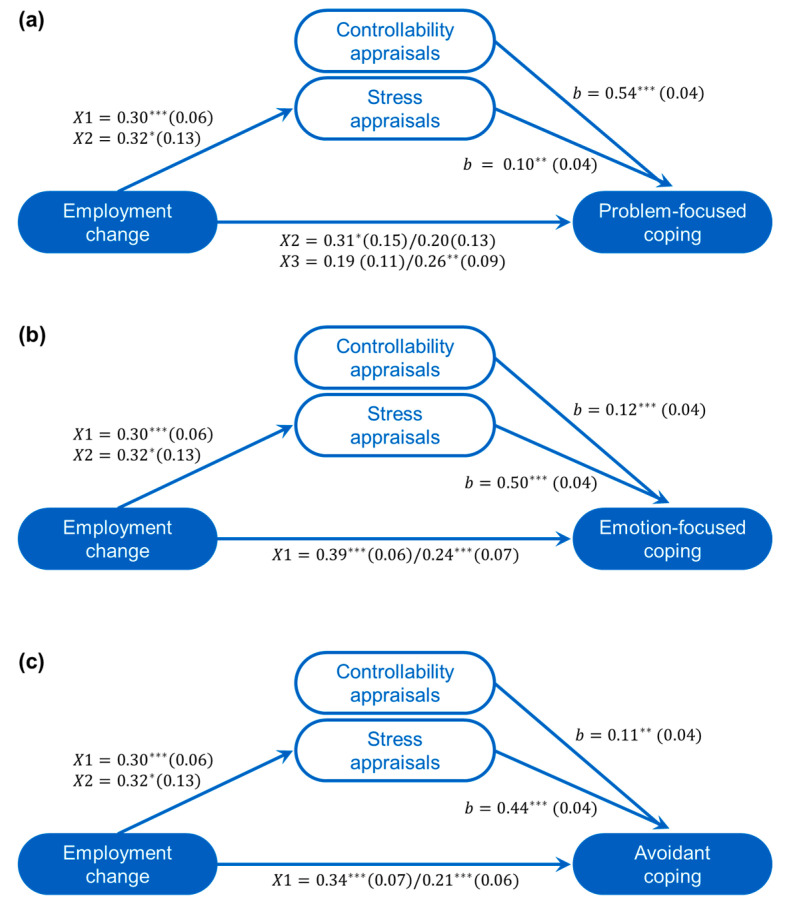
Stress appraisals from change in employment status predicted emotion-focused and avoidant coping strategies. Mediated models (unstandardized coefficients (*se*)) of the relationships between change of employment status and problem-focused (**a**), emotion-focused (**b**), and avoidant coping (**c**) as mediated through appraisals of controllability and stress (including total/direct effects between employment change and coping). X1 is the contrast between no change vs. change; X2 is the contrast between more hours vs. reduced hours/laid off; X3 is the contrast between reduced hours and being laid off; *b* is the unstandardized regression coefficient. * *p* < 0.05; ** *p* < 0.01; *** *p* < 0.001.

Analyses were conducted to assess whether COVID-19 appraisals mediated the relationships between change of employment status and the various coping strategies (each in a separate analysis). As seen in Figure 1, mediation analyses affirmed that employment change was a significant predictor of appraisals of stress (*R*^2^ = 0.070, *F*(3676) = 16.89, *p* < 0.001), but not controllability (*R*^2^ = 0.005, *F*(3676) = 1.23, *p* = 0.299) (as seen in the ANOVAs). In addition, when appraisals were included in the model, the direct relation between employment status and problem-focused coping remained significant (*R*^2^ = 0.013, *F*(3674) = 3.82, *p* = 0.010), reflecting a greater likelihood of using problem-focused coping when laid off (X3, *b* = 0.26 (*se* = 0.09), *p* = 0.005). 

In addition, the mediated relations between X1 (no change vs. change), *Effect* = 0.030 (*se* = 0.01), CI_.95_[0.006, 0.06], and X2 (more hours vs. reduced hours/laid off), *Effect* = 0.033 (*se* = 0.02), CI_.95_[0.001, 0.08] with problem-focused coping through stress appraisals were both significant (Figure 1a). Although controllability appraisals were significantly related to greater problem-focused coping, they did not mediate the relationship with change of employment status. 

The direct effect of employment change in relation to emotion-focused coping remained significant after controlling appraisals (*R*^2^ = 0.016, *F*(3674) = 4.79, *p* = 0.003), reflecting a greater likelihood of using emotion-focused coping when there was a change of employment status (X1, *b* = 0.24 (*se* = 0.07), *p* < 0.001). In addition, mediated relations between X1 (*Effect* = 0.150 (*se* = 0.03), CI_.95_[0.08, 0.22]) and X2 (more hours vs. reduced hours/laid off) (*Effect* = 0.164 (*se* = 0.07), CI_.95_[0.02, 0.31]) through stress appraisals were significant. Although it can be seen in Figure 1b that controllability appraisals were related to greater emotion-focused coping, they did not mediate the relationship with employment status.

Finally, when controlling appraisals, the direct effect of change of employment status in relation to avoidant coping remained significant (*R*^2^ = 0.021, *F*(3674) = 6.14, *p* < 0.001), reflecting a greater likelihood of avoidant coping being used when there was a change of employment (X1, *b* = 0.21 (*se* = 0.06), *p* = 0.001). Mediated relations between X1 (*Effect* = 0.129 (*se* = 0.03), CI_.95_[0.07, 0.19]) and X2 (*Effect* = 0.141 (*se* = 0.06), CI_.95_[0.02, 0.26]) through stress appraisals were also significant (Figure 1c). Once again, although appraisals of controllability were significantly related to greater avoidant coping, they were not a significant mediator.

Gender, income, living arrangements, and nationality were not significant moderators of these mediation models. Age, however, was a significant moderator of the pathways between X3 (reduced hours vs. laid off), stress appraisals, and each of problem-focused (*Index* = 0.002 (*se* = 0.001), CI_.95_[0.0001, 0.004]), emotion-focused (*Index* = 0.008 (*se* = 0.004), CI_.95_[0.001, 0.017]), and avoidant coping (*Index* = 0.007 (*se* = 0.004), CI_.95_[0.001, 0.014]). Specifically, age moderated the relationship between X3 and stress appraisals (*b* = 0.02 (*se* = 0.007), *p* = 0.017). Simple slope analyses conducted at 1 *SD* below and above the mean age indicated that this relation was not significant among younger participants (*b* = −0.11 (*se* = 0.13), *p* = 0.395), whereas among older participants, being laid off (relative to reduced hours) was appraised as especially stressful (*b* = 0.44 (*se* = 0.17), *p* = 0.011), which, in turn, was associated with greater coping efforts. Age did not moderate the direct relations between employment status and any of the coping strategies.

#### Summary

Change of employment status, and in particular reduced hours and being laid off, were associated with greater appraisals of the COVID-19 situation as stressful (but did not influence whether the situation was appraised as controllable) and elicited greater efforts to cope with the situation. Stress appraisals were also more likely to trigger greater coping efforts, particularly emotion-focused and avoidant strategies, whereas appraising the situation as controllable was more likely to be associated with problem-focused coping efforts. Older adults were especially likely to appraise being laid off as stressful (relatively to a reduction of working hours).

### 3.3. Were Stressor Appraisals and Coping Strategies Related to Current Affective States? 

#### 3.3.1. Positive Affect

Over and above the effects of change of employment status on positive affect (*R*^2^ = 0.014, *F*(3676) = 3.19, *p* = 0.023), both appraisals (*R*^2^*_change_*= 0.265, *F*(2674) = 123.97, *p* < 0.001) and coping strategies (*R*^2^*_change_* = 0.075, *F*(3671) = 26.10, *p* < 0.001) accounted for additional unique variance. The relationships between each of the predictors and positive affect are seen in Table 4. The strongest predictors of more positive affect were appraisals of the situation as controllable and the use of problem-focused coping strategies. Employment status change (in particular, being laid off) was uniquely, but more weakly associated with less positive affect, as were more emotional (stress) appraisals and both emotion-focused and avoidant coping strategies.

Age was a significant moderator of the relations between positive affect and both emotion-focused (*R*^2^ = 0.007, *F*(1676) = 5.105, *p* = 0.024) and avoidant coping (*R*^2^ = 0.009, *F*(1676) = 6.36, *p* = 0.012). Simple slope analyses indicated that as age increased (1 *SD* above the mean), there was an increasingly negative relationship between positive affect with both emotion-focused (*b* = −2.08 (*se* = 0.60), *p* < 0.001) and avoidant coping (*b* = −1.78 (*se* = 0.62), *p* = 0.005) at 1 *SD* below the mean (Emotion-focused, *b* = −0.31 (*se* = 0.53), *p* = 0.562; Avoidant, *b* = 0.32 (*se* = 0.56), *p* = 0.569). Likewise, income moderated the relationship between avoidant coping and positive affect (*R*^2^ = 0.007, *F*(1673) = 4.78, *p* = 0.029) such that at lower income levels, avoidant coping was associated with lower positive affect (*b* = −1.99 (*se* = 0.51), *p* < 0.001), whereas this relationship was not evident at higher income levels (*b* = −0.30 (*se* = 0.59), *p* = 0.613). None of the remaining demographic variables moderated the relationships between appraisals and coping predicators and positive affect.

#### 3.3.2. Negative Affect

Employment change (*R*^2^ = 0.056, *F*(3676) = 13.33, *p* < 0.001) was significantly related to negative affect. In addition, appraisals (*R*^2^*_change_* = 0.380, *F*(2674) = 226.79, *p* < 0.001) and coping (*R*^2^*_change_* = 0.084, *F*(3671) = 39.31, *p* < 0.001) contributed unique variance to predict negative affect. As seen in Table 4, the strongest predictors of more negative affect were appraisals of the situation as stressful and the use of emotion-focused and avoidant coping strategies (with problem-focused coping serving in a suppressor capacity). Employment status change did not demonstrate a unique relation with negative affect when appraisals and coping strategies were included in the equation (Table 4). None of the demographic variables moderated the relations between employment change, appraisals, and coping with negative affect.

#### 3.3.3. Summary

It appears that, not surprisingly, affective states during the COVID-19 pandemic were associated with changes of employment status, such that any change of status was associated with more negative affect. Moreover, being laid off was especially likely to be accompanied by diminished positive affect. However, most predictive of affect were appraisals and coping processes, with different elements of these processes being differentially linked to positive versus negative affective states. Positive affect was more likely to be reported among those who perceived the situation as controllable and used problem-focused coping efforts, whereas negative affect was more strongly linked to appraising their situations as stressful and endorsing emotion-focused coping strategies. This said, positive affect was negatively associated with emotion-focused and avoidant coping strategies among older, lower income participants.

### 3.4. Were Snacking Behaviors Associated with Stress-Related Affective States and Eating to with Stressors?

It was hypothesized that affective states would be predictive of snacking as a strategy for coping with affective changes. Before assessing these models, we examined whether the appraisal and general coping variables were related to coping by eating and snacking choices (Table 5). Stress appraisals and both emotion-focused and avoidant coping were related to coping by eating and with both salty and sweet snacking. Controllability appraisals and problem-focused coping were primarily related to wholesome snacking. These relationships suggest that the stress and coping variables that give rise to positive and negative affect might well underlie relationships between affect and eating choices.

#### 3.4.1. Eating Choices Associated with Positive Affect

**Figure 2:**
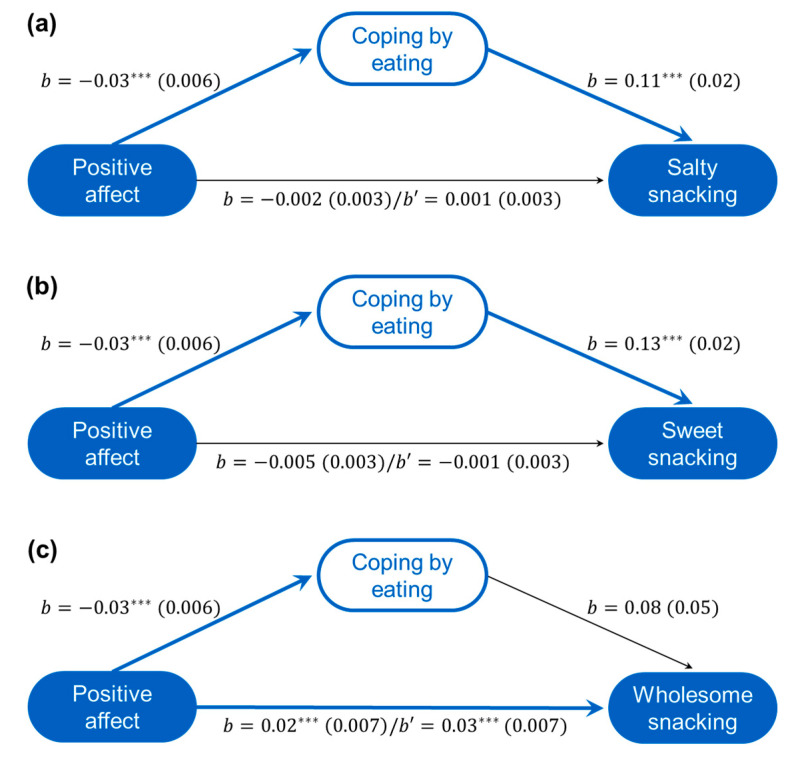
Positive affect associated with reduced eating to cope and increased wholesome snacking. Mediated models (unstandardized coefficients (*se*)) of the relationships between positive affect and salty (**a**), sweet (**b**), and wholesome snacking (**c**) as mediated through coping by eating (including total effect (*b*)/direct effect (*b’*) between affect and snacking). *** *p* < 0.001.

As seen in Figure 2, positive affect was a significant predictor of being less likely to cope by eating. Positive affect did not significantly predict salty (Figure 2a) or sweet snacking (Figure 2b) but was directly related to more wholesome snacking (Figure 2c). However, because coping by eating was related to greater salty and sweet snack consumption (Figure 2), the mediated models were significant (*Salty Effect* = −0.004 (*se* = 0.001), CI_.95_[−0.006, –0.002]; *Sweet Effect* = −0.004 (*se* = 0.001), CI_.95_[−0.006, −0.002]). Coping by eating did not mediate the relation between positive affect and whole food snacking (*Effect* = −0.003 (*se* = 0.002), CI_.95_[−0.006, 0.0004]).

The relationship between positive affect and being less likely to cope by eating was moderated by gender (*R*^2^ = 0.007, *F*(1661) = 5.31, *p* = 0.022) and is evident among females (*b* = −0.04 (*se* = 0.007), *p* < 0.001) but not males (*b* = −0.009 (*se* = 0.01), *p* = 0.460). Gender did not moderate the relations between eating to cope and snacking. Nonetheless, the mediated models predicting salty or sweet food choices were significant among females (*Salty Effect* = −0.006, *se* = 0.001, CI_.95_[−0.008, –0.003]; *Sweet Effect* = −0.006, *se* = 0.001, CI_.95_[−0.008, −0.003]) but not males (*Salty Effect* = −0.001, *se* = 0.002, CI_.95_[−0.004, 0.002]; *Sweet Effect* = −0.001, *se* = 0.001, CI_.95_[−0.004, 0.002]). The moderated mediation indices for both models were significant (*Salty Index* = 0.004, *se* = 0.002, CI_.95_[0.001, 0.008]; *Sweet Index* = 0.004, *se* = 0.002, CI_.95_[0.001, 0.008]). Gender did not alter the model predicting wholesome snacking.

In addition, the negative relation between positive affect and coping by eating was moderated by education (*R*^2^ = 0.009, *F*(1674) = 6.01, *p* = 0.015), and the negative relation was evident among those with some post-secondary education (*b* = −00.04 (*se* = 0.006), *p* < 0.001) but not those without (*b* = 0.00 (*se* = 0.02), *p* = 0.810). Thus, the mediated models predicting less salty or sweet food consumption were significant among those with more education (*Salty Effect* = −0.004, *se* = 0.001, CI_.95_[−0.007, −0.002]; *Sweet Effect* = −0.005, *se* = 0.001, CI_.95_[−0.007, −0.003]) but not those with less education (*Salty Effect* = 0.00, *se* = 0.002, CI_.95_[−0.003, 0.004]; *Sweet Effect* = 0.00, *se* = 0.002, CI_.95_[−0.004, 0.005]). The moderated mediation indices for both models was significant (*Salty Index* = −0.005, *se* = 0.002, CI_.95_[−0.010, −0.001]; *Sweet Index* = −0.005, *se* = 0.003, CI_.95_[−0.011, −0.001]). Education did not alter the model predicting wholesome snacking. 

None of change of employment status, age, income, living arrangement, or national location moderated any of the mediation pathways in the relations between positive affect and snacking. The moderating role of BMI was also assessed and found to not be significant.

#### 3.4.2. Eating Choices Associated with Negative Affect

**Figure 3:**
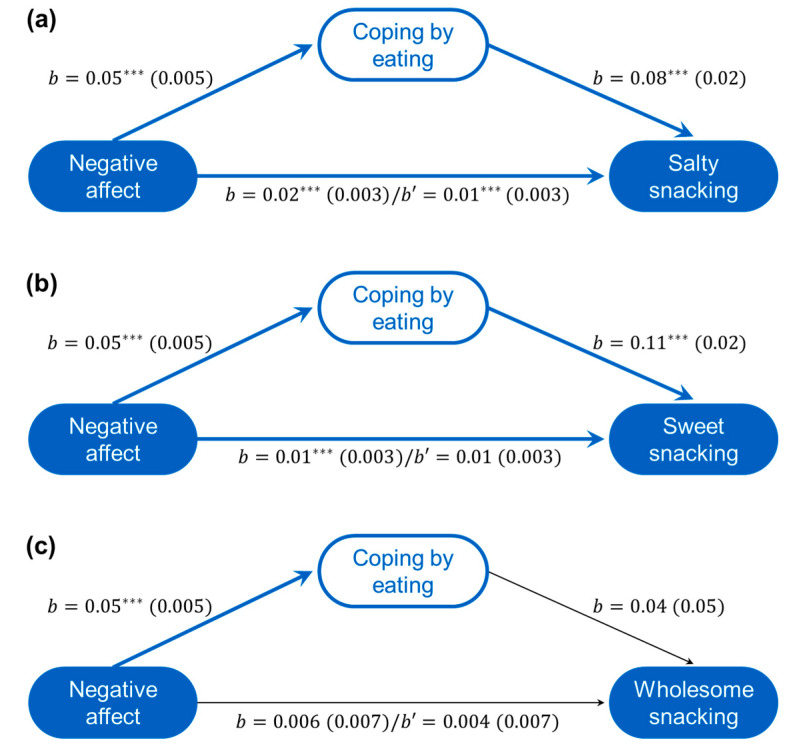
Negative affect predicted coping by eating and unhealthy snacking. Mediated models (unstandardized coefficients (*se*)) of the relationships between negative affect and salty (**a**), sweet (**b**), and wholesome snacking (**c**) as mediated through coping by eating (including total effect (*b*)/direct effect (*b’*) between affect and snacking. *** *p* < 0.001.

As seen in Figure 3, negative affect was a significant predictor of coping by eating and was a significant direct predictor of eating more salty and sweet snacks. In both instances, the mediating role of coping by eating was significant (*Salty Effect* = 0.004 (*se* = 0.001), CI_.95_[0.002, 0.006]; *Sweet Effect* = 0.005 (*se* = 0.001), CI_.95_[0.003, 0.007]). Neither negative affect (*b* = 0.004 (*se* = 0.007), *p* = 0.568) nor coping by eating was related to more whole food snacking (*b* = 0.04 (*se* = 0.05), *p* = 0.449).

The relation between negative affect and coping by eating was moderated by education (*R*^2^ = 0.021, *F*(1674) = 16.12, *p* < 0.001) and was evident among those with some post-secondary education (*b* = 0.05 (*se* = 0.006), *p* < 0.001) but not those without (*b* = −0.004 (*se* = 0.01), *p* = 0.796). Thus, the mediated relations predicting less salty or sweet food consumption through coping by eating was significant among those with more education (*Salty Effect* = 0.005, *se* = 0.001, CI_.95_[0.003, 0.008]; *Sweet Effect* = 0.006, *se* = 0.001, CI_.95_[0.004, 0.009]) but not those with less education (*Salty Effect* = 0.00, *se* = 0.002, CI_.95_[−0.003, 0.003]; *Sweet Effect* = −0.00, *se* = 0.002, CI_.95_[−0.004, 0.003]). The moderated mediation indices for both models was significant (*Salty Index* = 0.005, *se* = 0.002, CI_.95_[0.002, 0.010]; *Sweet Index* = 0.007, *se* = 0.002, CI_.95_[0.003, 0.011]). Education did not alter the model predicting wholesome snacking.

None of change of employment status, age, gender, income, living arrangement, or national location moderated any of the mediation pathways in the relations between negative affect and snacking. The moderating role of BMI was also assessed and found to not be significant.

#### 3.4.3. Summary

It appears that positive affect was associated with less likelihood of eating to cope, which in turn was associated with lower inclination to engage in salty or sweet snacking. This was particular the case among women and those with more education. In contrast, negative affect was more likely to trigger eating to cope and, hence, greater salty and sweet snacking and, once again, the mediating role of eating to cope was especially evident among those with more education. None of the mediated relations between affect, eating to cope or snacking choices was moderated by change of employment status.

## 4. Discussion

This study aimed to determine the variables underlying the affect associated with COVID-19 stress reactions and unhealthy eating during the pandemic. One of the most prominent consequences of the outbreak was home confinement, which resulted in drastic shifts of lifestyles and employment status. As seen in other studies, employment change involving job loss or reduced work hours during the COVID-19 outbreak was widespread [51,52] disproportionately affecting younger and lower income participants [53,54]. Such changes were appraised as stressful and uncontrollable; individuals tended to avoid the issue or use emotion-based strategies to cope with distress. Employment change also directly predicted negative affect, which was associated with unhealthy snacking. Eating is an emotion-based coping strategy [55], and during the COVID-19 outbreak, individuals were found to cope by eating, and in particular, they tended to eat more salty or sweet processed snacks but not wholesome snacks, such as fruit or vegetables. In contrast, positive affect was inversely related to emotional eating, and was directly related to consumption of wholesome snacks.

Many individuals lost their jobs or worked fewer hours during the COVID-19 outbreak, as stay-at-home orders forced the closure of businesses and workplaces. The extended period of uncertainty and job insecurity, a known work stressor [56], contributed to worsened mental health states and increased reports of anxiety and depression [5,41,42]. As previously reported [53,54] and in our study, females and younger workers, as well as those living in shared accommodations or lower income households were more likely to be laid off. While our survey did not identify worker occupations, findings from other research suggests that this demographic profile may be attributable to the prevalence of young people and women in the restaurant or service industry [57,58,59], which was most adversely impacted by the COVID-19 outbreak [60,61]. As expected, those reporting a reduction or loss of employment were more likely to appraise the COVID-19 situation as stressful and reported higher negative and lower positive affect. It has been suggested that financial pressure and fear of not working may drive the relationship between job insecurity and mental state [5,62], and indeed, lower income individuals vulnerable to employment loss reported worsened mood in the present study.

In order to understand mood outcomes linked to COVID-19 stressors, we assessed subjective appraisals and coping strategies. Workers who experienced reduced work or loss of work were more likely to appraise their situation to be stressful and out of their control. As expected, elevated stress appraisals were, in turn, related to avoidant or emotion-focused coping strategies, whereas appraising the COVID-19 situation to be controllable was accompanied by greater use of problem-focused coping strategies. The uncertainty surrounding the COVID-19 situation may make it difficult to prepare or plan job searches, thus hindering the use of problem-focused coping strategies [44].

Eating is a form of emotion-based coping to deal with stressors [55,63]. Indeed, our data showed a strong correlation between coping by eating with appraisals of stress, together with emotion-focused and avoidant coping strategies. An individual may use food to cope with their internal emotions, as well as with external stressors, such as change of employment status [28]. Our findings are consistent with numerous studies from Europe [7,34,35,36] and North America [3,6,64] indicating that during the COVID-19 outbreak increased feelings of depression, anxiety, and stress, and a worsened mental state was associated with emotional eating. Indeed, COVID-19 appeared to have a clear relationship to eating behaviors, particularly eating more snack-type foods [3,11]. In this regard, individuals who appraised their situation to be stressful or who experienced greater negative mood were more likely to consume both salty and sweet snacks. We do not have retrospective reports of food intake prior to the COVID-19 outbreak and, hence, cannot ascertain whether these food habits were ascribed to COVID-19. However, given that self-reports typically lead to inaccurate reporting, particularly of unhealthy snacks [65], the value of retrospective data is questionable. The fact is that in the present investigation, employment status and coping methods were tied to snacking and specific types of foods consumed. Moreover, greater perceived distress was associated with lower consumption of healthy foods like fruits or vegetables [27]. This is in line with previous research indicating that negative mood is associated with hedonic food consumption, whereas positive mood is associated with eating fewer hedonic foods [32,33]. It should be noted that although stress can shift eating behavior to favor snacks over meal-type foods, it does not necessarily mean that individuals are overeating [26].

Change in the amount of food consumed is commonly linked to baseline dieting status. Individuals who ordinarily restrict their diet may be more likely to overeat when experiencing increased stress, particularly as they lose control over their eating [26]. Females are more frequent dieters than are males, and more susceptible to emotional eating [66,67]. In fact, females were shown to eat more high caloric foods during home confinement [34]. We focused on the types of food rather than the amount of food consumed and found that female participants reported higher consumption of healthy, but not unhealthy, snacks. That said, females are more likely to under- and/or mis-report their food intake [68], which may contribute to our findings. Furthermore, unhealthy food consumption associated with mood states varies with basal body weight. Specifically, underweight individuals eat less than normal or overweight individuals when in a negative mood state, but may eat more when in a positive mood state [55]. In contrast, overweight individuals tend to overeat during negative mood states [29,30]. Individuals with higher BMI may be more restrained eaters [69] and, hence, may have exhibited less control over their eating during home confinement [7]. We similarly found that high BMI was related to lower whole food snacking, but BMI did not mediate the relationships between stress and coping or mood and snacking behaviors.

### Limitations

There are several caveats that ought to be mentioned with respect to the findings of the present investigation. As alluded to earlier, it would have been ideal to have data available concerning changes of snack intake relative to the pre-COVID period. Nevertheless, the present findings speak to the links between snack choices and employment change, mood state, and coping methods. A second limitation of our investigation concerns the lack of a representative sample given that participants were largely recruited through various digital or social media platforms. For instance, the present sample were highly educated and also seemed to have a higher household income. Finally, although we have ascribed the altered eating profiles to employment related distress and coping factors, it is possible that several other factors (e.g., more time at home, boredom) contributed to unhealthy eating.

## 5. Conclusions

Although the pandemic gave rise to multiple stressors (e.g., fear of contamination, social isolation), financial challenges including employment change have been amongst the most distressing. We showed that distress from employment change was associated with altered mood and unhealthy eating, particularly for carbohydrate-rich foods. Indeed, greater perceived stress or more negative mood was associated with emotional eating and increased consumption of salty or sweet snacks. Poor food choices and increased snack consumption [70], especially when combined with reduced physical activity [11], can lead to weight gain, which was reported in a quarter of people sampled during the COVID-19 outbreak [13]. The indirect impact of COVID-19 includes shifts in food choices, which can be detrimental to overall health. This is concerning given the prevalence of overweight and obesity within the population, and emerging evidence has indicated that obesity or obesity-related diseases increase the risk for more severe outcomes among those infected with COVID-19 [20,22,23].

Government agencies have increasingly been advising individuals to adopt public health measures (e.g., wearing face masks, handwashing, physical distancing), and there has also been concern regarding potential mental health challenges. Much less attention seems to have been devoted to the indirect impact of the pandemic on unhealthy behaviors, such as those related to food intake. In considering factors important for emergency preparedness, a series of recommendations were made concerning those factors that favor resilience during such situations and the recovery period that follows [71]. Essential to this is that individuals have social support available as a potent coping resource prior to the emergency or, failing this, that effective support networks can be readily established. The present findings are consistent with this view and point to the need to address health-related behaviors beyond those that focus only on psychological disturbances.

## Figures and Tables

**Table 1:** Description of participant characteristics.

Variable	Number of Participants (%)
**Gender**	
Female	510 (75.0%)
Male	155 (22.8%)
Other (e.g., transgender, non-binary)	15 (2.2%)
**Cultural affiliation**	
White and/or Euro-Caucasian	523 (76.9%)
Black and/or African	29 (4.3%)
Asian (West, South, East, or Southeast)	121 (17.8%)
Latin American and Caribbean	29 (4.3%)
Indigenous	12 (1.7%)
Other	5 (0.7%)
**Relationship status**	
Single, not seeing anyone	211 (31.0%)
In a relationship	146 (21.5%)
Cohabitating/Married	288 (42.4%)
Separated/Divorced	27 (4.0%)
Widowed	8 (1.2%)
**Household income**	
Under $15,000 (1) ^1^	35 (5.1%)
$15,000–$29,999 (2)	61 (9.0%)
$30,000–$44,999 (3)	55 (8.1%)
$45,000–$59,999 (4)	76 (11.2%)
$60,000–$74,999 (5)	76 (11.2%)
$75,000–$89,999 (6)	77 (11.3%)
$90,000–$104,999 (7)	69 (10.1%)
$105,000 or more (8)	228 (33.5%)
**Employment status**	
Employed Part-time	128 (18.8%)
Employed Full-time	286 (42.1%)
Self-employed	50 (7.4%)
Unemployed	147 (21.6%)
Retired	54 (7.9%)
Other	15 (2.2%)
**Location**	
Canada	559 (82.2%)
United States	121 (17.8%)
**Living arrangement**	
Living alone	95 (14.0%)
Living with others	408 (60.0%)
Living with others and children	156 (22.9%)
Living alone and children	21 (3.1%)
**Education**	
High school or less	94 (13.8%)
Post-secondary education	584 (85.9%)
Other	2 (0.3%)

^1^ numeral in bracket correspond to the categorical value assigned to income for statistical analysis.

**Table 2:** Means (*SD*)/*n* (percentage of employment categories) associated with change of employment status.

	No Change (*n* = 389)	More Hours (*n* = 34)	Reduced Hours (*n* = 97)	Laid Off (*n* = 160)
**Age ^1^**	40.48 (16.01) _a_	38.23 (15.42) _a,b_	34.46 (12.68) _b_	29.09 (13.19) _c_
**Income ^1,2^**	5.77 (2.26) _a_	6.35 (2.39) _a_	5.01 (2.22) _b_	5.29 (2.37) _a,b_
**Gender ^3^**				
Female	290 (56.9%)	26 (5.3%)	60 (11.8%)	133 (26.1%)
Male	93 (60.0%)	6 (3.9%)	34 (21.9%)	22 (14.2%)
**Education ^3^**				
High school or less	37 (39.4%)	7 (7.4%)	7 (7.4%)	43 (45.7%)
Some post-secondary	350 (59.9%)	27 (4.6%)	90 (15.4%)	117 (20.0%)
**Living arrangement ^3^**				
Alone	73 (76.8%)	4 (4.2%)	8 (8.4%)	10 (10.5%)
With others	205 (50.2%)	21 (5.1%)	59 (14.5%)	123 (30.1%)
Others with children	100 (64.1%)	7 (4.5%)	26 (16.7%)	23 (14.7%)
Alone with children	11 (52.4%)	2 (9. 5%)	4 (19.0%)	4 (19.0%)
**National location ^3^**				
Canada	308 (55.1%)	28 (5.0%)	68 (12.2%)	155 (27.7%)
United States	81 (66.9%)	6 (5.0%)	29 (24.0%)	5 (4.1%)

_a,b,c_ Columns with different subscripts differ significantly from one another at *p* < 0.05. ^1^ Analyzed with ANOVA. ^2^ Income codes reflect brackets of annual income ranging from 1 (<$15,000) to 8 (>$105,000). Thus, sample income means in each column were between 5 ($60,000–$74,999) and 6 ($75,000–$89,999). ^3^ Analyzed with the chi-square test.

**Table 3:** Means (SD) of model variables as a function of change of employment status.

	No Change (*n* = 389)	More Hours (*n* = 34)	Reduced Hours (*n* = 97)	Laid Off (*n* = 160)	η^2^
**Appraisals**					
Stress	2.61 (0.73) _a_	2.69 (0.77) _a_	2.97 (0.73) _b_	3.05 (0.73) _b_	0.070 ***
Controllability	3.10 (0.74)	3.00 (0.74)	3.20 (0.63)	3.04 (0.72)	0.005
**Coping**					
Problem-focused	3.13 (0.82) _a,b_	2.91 (0.84) _a_	3.13 (0.78) _a,b_	3.31 (0.83) _b_	0.013 *
Emotion-focused	2.22 (0.82) _a_	2.56 (0.96) _b_	2.62 (0.87) _b_	2.66 (0.80) _b_	0.057 ***
Avoidant	2.57 (0.79) _a_	2.74 (0.88) _a,b_	2.94 (0.79) _b_	3.03 (0.76) _b_	0.065 ***
Eating	2.52 (1.25)	2.77 (1.33)	2.87 (1.24)	2.96 (1.24)	0.024 ***
**Affect**					
Positive	27.69 (8.44)	28.21 (9.56)	29.41 (8.21)	26.16 (7.93)	0.014 *
Negative	18.22 (8.01) _a_	22.09 (9.43) _b_	21.01 (8.69) _a,b_	23.01 (9.52) _b_	0.056 ***
**Snacking**					
Salty	1.68 (0.66) _a_	1.88 (0.74) _a,b_	2.03 (1.00) _b_	1.78 (0.75) _a,b_	0.026 ***
Sweet	1.79 (0.66)	1.86 (0.82)	2.01 (0.78)	1.90 (0.70)	0.013 *
Wholesome	3.67 (1.63)	3.69 (1.53)	3.49 (1.57)	3.63 (1.53)	0.002

_a,b_ Columns with different subscripts differ significantly from one another at *p* < 0.05; * *p* < 0.05; *** *p* < 0.001.

**Table 4:** Regression coefficients predicting positive and negative affect based on final step statistics.

	Positive Affect				Negative Affect			
	*b*	*se*	*Beta*	*r*	*b*	*se*	*Beta*	*r*
**Employment change**								
No change vs. change	0.43	0.15	0.10 **	−0.01	0.16	0.14	–0.05	0.23 ***
More hours vs. reduced/laid off	−0.36	0.42	−0.03	−0.02	−0.65	0.38	−0.05	0.11 **
Reduced hours vs. laid off	−1.44	0.44	−0.11 ***	−0.12 ***	0.74	0.40	0.05	0.11 **
**Appraisals**								
Stress	−1.21	0.41	−0.11 **	−0.21 ***	5.47	0.37	0.47 ***	0.65 ***
Controllability	4.22	0.42	0.36 ***	0.49 ***	−0.59	0.38	−0.05	−0.10 *
**Coping**								
Problem-focused	2.96	0.38	0.29 ***	0.37 ***	−1.22	0.34	−0.11 ***	0.01
Emotion-focused	−1.58	0.40	−0.16 ***	−0.16 ***	2.82	0.36	0.28 ***	0.54 ***
Avoidant	−1.08	0.42	−0.11 *	−0.12 **	1.26	0.38	0.12 ***	0.45 ***

*b*, unstandardized regression coefficient; *se*, standard error; *Beta*, standardized coefficient; *r*, zero-order correlation. * *p* < 0.05; ** *p* < 0.01; *** *p* < 0.001.

**Table 5:** Correlations between appraisals and general coping strategies with coping by eating and snacking behaviors (*n* = 680).

	Coping by Eating	Snacking		
		Salty	Sweet	Wholesome
**Appraisals**				
Stressful	0.33 ***	0.14 ***	0.17 ***	0.01
Controllable	0.03	−0.02	0.01	0.13 ***
**Coping**				
Problem-focused	0.07	0.004	0.04	0.24 ***
Emotion-focused	0.31 ***	0.15 ***	0.17 ***	0.09 *
Avoidant	0.43 ***	0.17 ***	0.18 ***	0.06

* *p* < 0.05; *** *p* < 0.001.

## Supplementary Materials

The following are available online at https://www.mdpi.com/2072-6643/12/12/3803/s1, Table S1: Distribution of recruitment methods for participants included in the survey (*n* = 680), Table S2: Number of participants recruited but excluded from the survey (*n* = 101), Table S3: Highest level of education completed by participants (*n* = 680), Table S4: Means (*SD*)/*n* (percent of participant) associated with participants who experienced “No change” in employment.
