# Supplementary material for: Piece of Cake: Coping with COVID-19
Source: Nutrients. 2020 Dec 11;12(12):3803. doi: 10.3390/nu12123803 (PMC7764173; doi:10.3390/nu12123803)
Supplement: Supplementary file 1 [file nutrients-12-03803-s001.pdf]

Table S1. Distribution of recruitment methods for participants included in the survey (*n* = 680).

Recruitment method	Number of participants (%)
Email	283 (41.6%)
Social media (e.g., Facebook, Instagram, Twitter)	157 (23.1%)
MTurk	91 (13.4%)
Research website	6 (0.9%)
Online classifieds (e.g., Reddit, Kijij)	1 (0.1%)
Other	119 (17.5%)
Did not specify	23 (3.4%)

Table S2. Number of participants recruited but excluded from the survey (n = 101).

Reason for exclusion	Number of participants (%)
One or more questions unanswered	43 (42.6%)
Selected *Other* when asked: *How did your employment status change as a result of COVID-19?*	28 (27.7%)
Withdrew from survey	16 (15.8%)
BMI is < 15 or > 55	11 (10.9%)
Survey was completed in under < 5 minutes	2 (2.0%)
Responded *No* when asked: *I provided honest, high-quality answers to the survey questions*	1 (1.0%)

Table S3. Highest level of education completed by participants (*n* = 680).

Education level	Number of participants (%)
**High school or less**	
No or some high school completed	3 (0.4%)
High school diploma	91 (13.4%)
**Post-secondary education**	
College/university credit, no diploma	112 (16.5%)
Trade/technical/vocational training	25 (3.7%)
Associate degree	17 (2.5%)
Bachelor’s degree	247 (36.3%)
Master’s degree	96 (14.1%)
Professional degree	25 (3.7%)
Doctorate degree	47 (6.9%)
**Other**	17 (2.5%)

Table S4. Means (SD)/ *n* (percent of participant) associated with participants who experienced "No change" in employment.

	**Employed (*n* = 266)**	**Unemployed (*n* = 65)**	**Retired (*n* = 51)**	**Other [4] (*n* = 7)**
**Age [1]**	38.31 (12.04) $_{a}$	28.71 (11.76) $_{b}$	68.14 (7.53) $_{c}$	37.86 (9.58) $_{a,b}$
**Income [1, 2]**	5.94 (2.23)	5.28 (2.58)	5.38 (1.82)	6.43 (2.07)
**Gender [3]**				
Female	188 (70.7%)	51 (78.5%)	44 (86.3%)	7 (100%)
Male	75 (28.2%)	11 (16.9%)	7 (13.7%)	0 (0%)
**Education [3]**				
High school or less	19 (7.1%)	17 (26.2%)	1 (2%)	0 (0%)
Some post-secondary	245 (92.1%)	48 (73.8%)	50 (98%)	7 (100%)
**Living arrangement [3]**				
Alone	46 (17.3%)	7 (10.8%)	20 (39.2%)	0 (0%)
With others	133 (50%)	51 (78.5%)	21 (41.2%)	0 (0%)
Others with children	78 (29.3%)	6 (9.2%)	10 (19.6%)	6 (85.7%)
Alone with children	9 (3.4%)	1 (1.5%)	0 (%)	1 (14.3%)
**National location [3]**				
Canada	194 (72.9%)	60 (92.3%)	47 (92.2%)	7 (100%)
United States	72 (27.1%)	5 (7.7%)	4 (7.8%)	0 (0%)

a,b,c Columns with different subscripts differ significantly from one another at $p < 0.05$.

1 Analyzed with ANOVA.

2 Income codes reflect brackets of annual income ranging from 1 (< $15,000) to 8 (> $105,000). Thus, sample income means in each column were between 5 ($60,000–$74,999) and 6 ($75,000–$89,999).

3 Analyzed with the chi-square test.

4 Includes individuals specifying long term leave, e.g., disability or maternity leave.
